# Supplementary material for: The proton motive force maintains mtDNA euploidy by balancing mtDNA replication with cell proliferation
Source: bioRxiv. 2025 Oct 27:2025.10.27.684790. Preprint. [Version 1] doi: 10.1101/2025.10.27.684790 (PMC12636550; doi:10.1101/2025.10.27.684790)
Supplement: Supplement 1 [file NIHPP2025.10.27.684790v1-supplement-1.pdf]

bioRxiv preprint doi: https://doi.org/10.1101/2025.10.27.684790; this version posted October 27, 2025. The copyright holder for this preprint (which was not certified by peer review) is the author/funder, who has granted bioRxiv a license to display the preprint in perpetuity. It is made available under aCC-BY 4.0 International license.

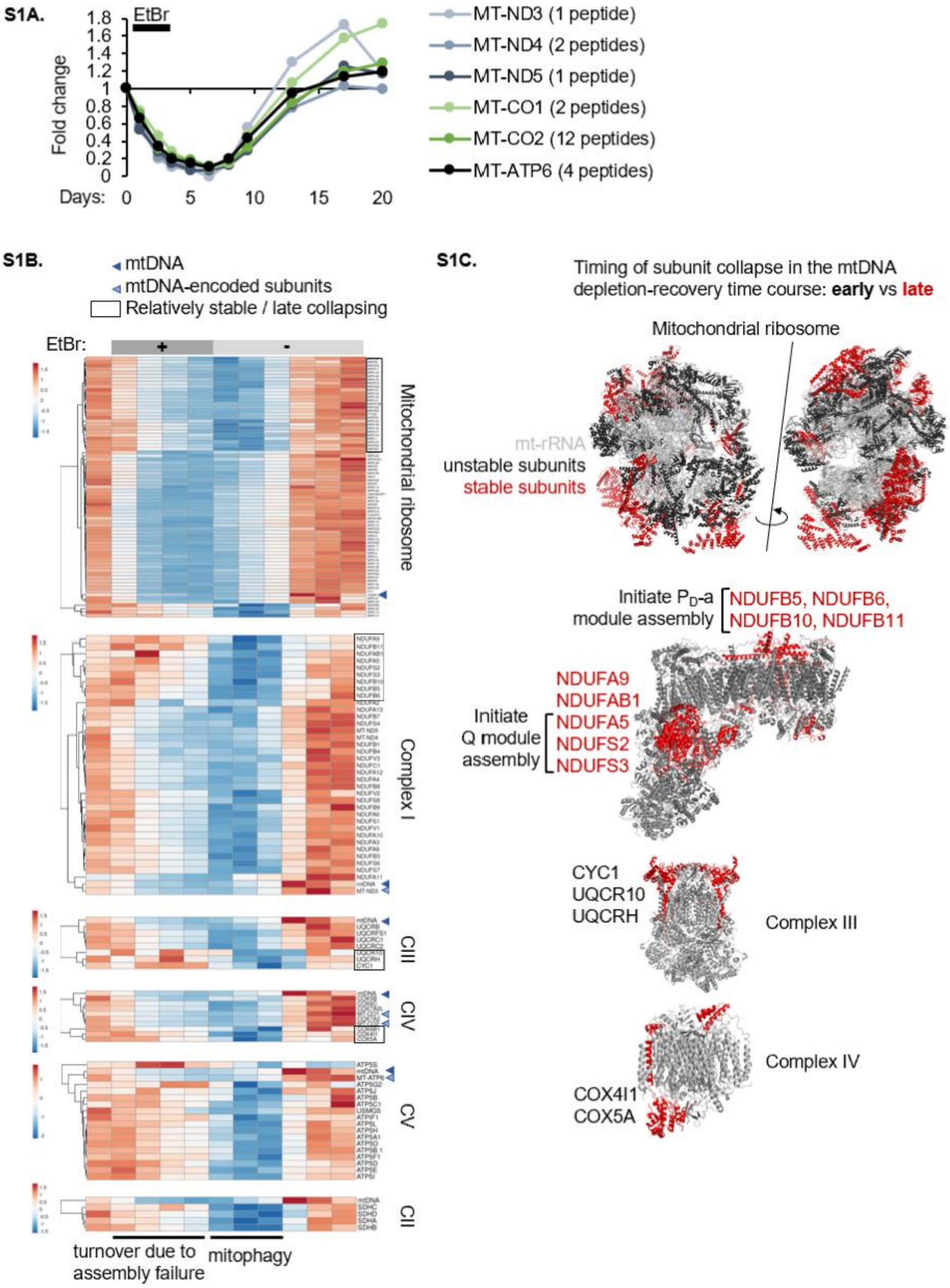

Figure S1: Temporal analysis of subunit loss upon mtDNA depletion in the context of the 3D structure of each complex

(A) Levels of mtDNA-encoded proteins detected by TMT proteomics during mtDNA depletion-recovery. **(B)** Heat map of all proteins detected in each indicated complex. mtDNA levels and mtDNA-encoded proteins are indicated by arrows. Black box indicates relatively stable subunits, which collapse later, as revealed by hierarchical clustering. **(C)** Subunits of all dual genome-encoded complexes color-coded on the 3D structure by timing of collapse in the mtDNA depletion-recovery system (early vs late collapsing).

bioRxiv preprint doi: https://doi.org/10.1101/2025.10.27.684790; this version posted October 27, 2025. The copyright holder for this preprint (which was not certified by peer review) is the author/funder, who has granted bioRxiv a license to display the preprint in perpetuity. It is made available under aCC-BY 4.0 International license.

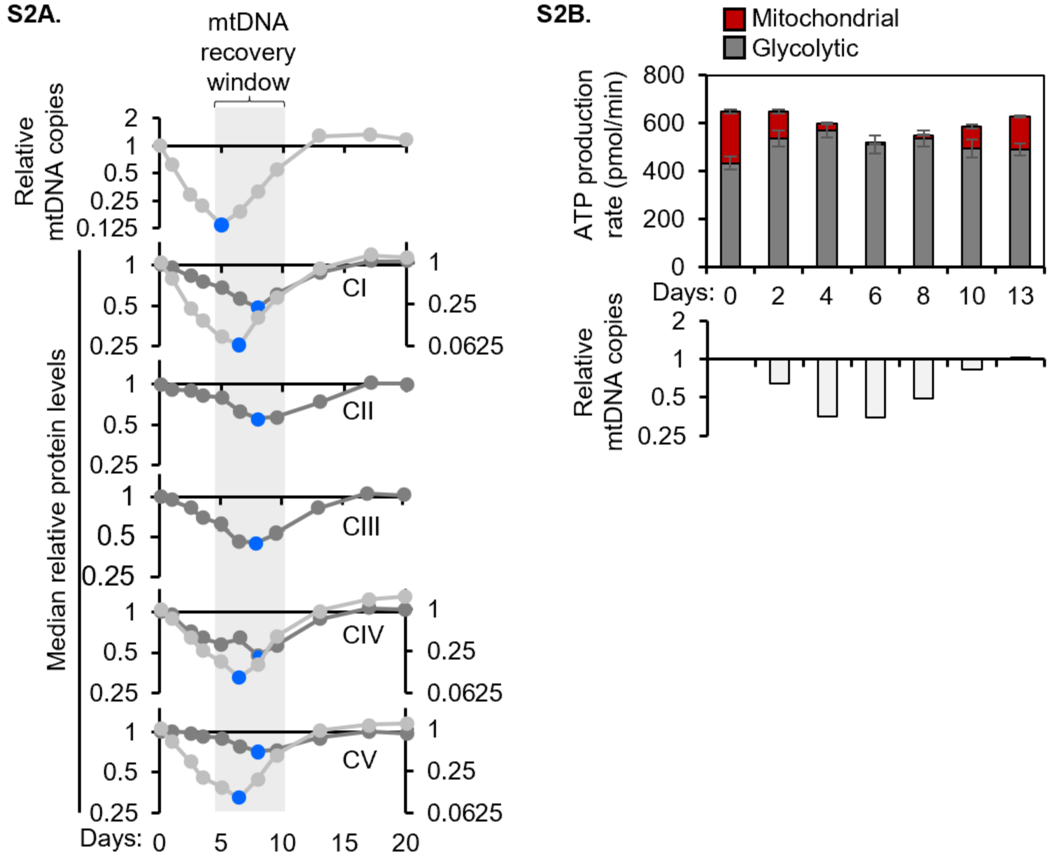

Subunits detected:

	CI	CII	CIII	CIV	CV
mtDNA-encoded:	3	n/a	0	2	1
Nuclear-encoded:	33	4	7	7	16

● Lowest level during time course

Figure S2. OXPHOS remains diminished during mtDNA recovery
(A) Median levels of all OXPHOS complex subunits detected by TMT proteomics. MtDNA- and nuclear-encoded subunits are shown separately. A blue dot is used to indicate the lowest level of each entity during the mtDNA depletion-recovery timecourse. (**B**) Stacked bar plots of mitochondrial and glycolytic ATP production rates measured in at least three replicates during a representative mtDNA depletion-recovery timecourse and mtDNA copy number measured by qPCR at the corresponding timepoints.

bioRxiv preprint doi: https://doi.org/10.1101/2025.10.27.684790; this version posted October 27, 2025. The copyright holder for this preprint (which was not certified by peer review) is the author/funder, who has granted bioRxiv a license to display the preprint in perpetuity. It is made available under aCC-BY 4.0 International license.

S3A.

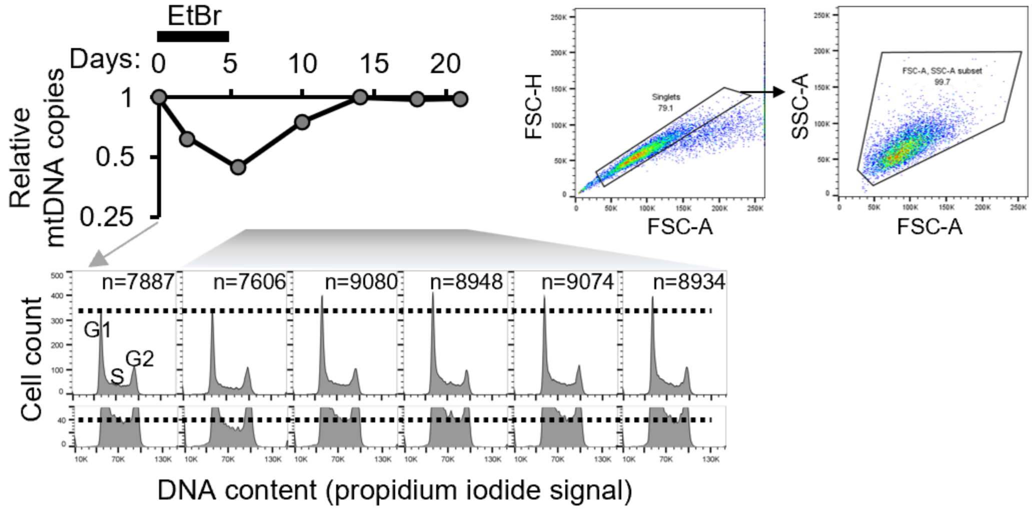

S3B.

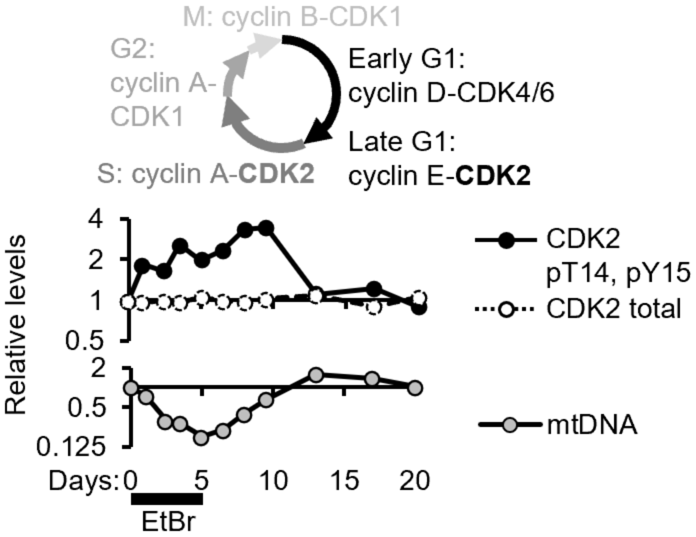

Figure S3: Cell cycle analysis during mtDNA depletion and recovery
(A) Cell cycle analysis by flow cytometry of asynchronous cultures from day 0 and all timepoints during mtDNA copy number recovery. Bottom panel is zoomed to show S phase, horizontal lines are drawn to aide visualization of the slight up-tick in G1 and slight decrease in S phase on day 5. Gates used to select single cells and specify side and forward scatter areas are shown. **(B)** Schematic of the cell cycle. Quantification of mtDNA copy number (qPCR), CDK2 protein, and CDK2 phospho-peptide capturing the inhibitory phosphosites pT14, pY15 (TMT proteomics).

bioRxiv preprint doi: https://doi.org/10.1101/2025.10.27.684790; this version posted October 27, 2025. The copyright holder for this preprint (which was not certified by peer review) is the author/funder, who has granted bioRxiv a license to display the preprint in perpetuity. It is made available under aCC-BY 4.0 International license.

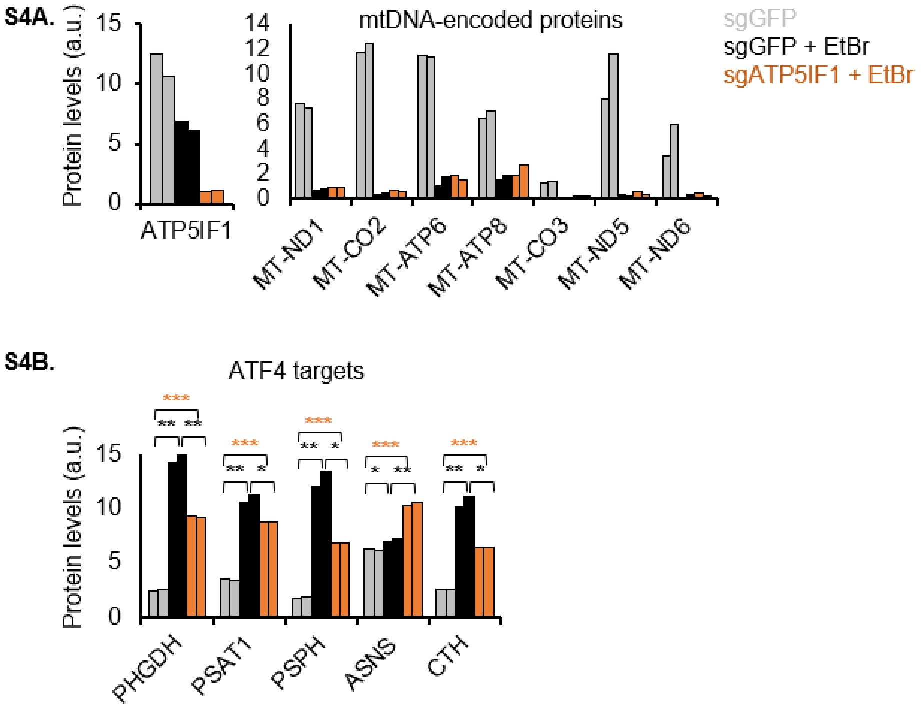

Figure S4: ATF4 response persists in mtDNA-depleted ATP5IF1 KO cells.
Protein levels of **(A)** ATP5IF1, mtDNA-encoded proteins, and **(B)** ATF4 targets in control vs ATP5IF1 KO treated with EtBr for 14 days. Asterisks denote p value from a two-tailed Students' t-test *<0.05, **<0.01, ***<0.001.
